# TGF-βRII Knock-down in Pancreatic Cancer Cells Promotes Tumor Growth and Gemcitabine Resistance. Importance of STAT3 Phosphorylation on S727

**DOI:** 10.3390/cancers10080254

**Published:** 2018-07-31

**Authors:** Vincent Drubay, Nicolas Skrypek, Lucie Cordiez, Romain Vasseur, Céline Schulz, Nihad Boukrout, Belinda Duchêne, Lucie Coppin, Isabelle Van Seuningen, Nicolas Jonckheere

**Affiliations:** 1 INSERM, UMR-S1172, Jean Pierre Aubert Research Center, “Mucins, Epithelial Differentiation and Carcinogenesis” Team, rue Polonovski, 59045 Lille CEDEX, France; v.drubay54@gmail.com (V.D.); nicolas.skrypek@gmail.com (N.S.); lucie.cordiez@gmail.com (L.C.); romain.vasseur@inserm.fr (R.V.); celine.schulz@univ-lille.fr (C.S.); nihad.boukrout@inserm.fr (N.B.); belinda.duchene@inserm.fr (B.D.); lucie.coppin@inserm.fr (L.C.); isabelle.vanseuningen@inserm.fr (I.V.S.); 2 UMR-S 1172—JPArc, Université Lille Nord de France, 1 Place de Verdun, 59045 Lille CEDEX, France; 3 Centre Hospitalier Régional et Universitaire de Lille, Place de Verdun, 59037 Lille CEDEX, France; 4 CNRS, UMR 8576-UGSF-Unité de Glycobiologie Structurale et Fonctionnelle, Université de Lille, F 59000 Lille, France

**Keywords:** TGF-βRII receptor, STAT3, metastasis, gemcitabine, ABC transporters, pancreas

## Abstract

Pancreatic adenocarcinoma (PDAC) is one of the most deadly cancers in the Western world because of a lack of early diagnostic markers and efficient therapeutics. At the time of diagnosis, more than 80% of patients have metastasis or locally advanced cancer and are therefore not eligible for surgical resection. Pancreatic cancer cells also harbour a high resistance to chemotherapeutic drugs such as gemcitabine that is one of the main palliative treatments for PDAC. Proteins involved in TGF-β signaling pathway (SMAD4 or TGF-βRII) are frequently mutated in PDAC (50–80%). TGF-β signalling pathway plays antagonistic roles during carcinogenesis by initially inhibiting epithelial growth and later promoting the progression of advanced tumors and thus emerged as both tumor suppressor and oncogenic pathways. In order to decipher the role of TGF-β in pancreatic carcinogenesis and chemoresistance, we generated CAPAN-1 and CAPAN-2 cell lines knocked down for TGF-βRII (first actor of TGF-β signaling). The impact on biological properties of these TGF-βRII-KD cells was studied both in vitro and in vivo. We show that TGF-βRII silencing alters tumor growth and migration as well as resistance to gemcitabine. TGF-βRII silencing also leads to S727 STAT3 and S63 c-Jun phosphorylation, decrease of MRP3 and increase of MRP4 ABC transporter expression and induction of a partial EMT phenotype. These markers associated with TGF-β signaling pathways may thus appear as potent therapeutic tools to better treat/manage pancreatic cancer.

## 1. Introduction

Pancreatic cancers (PC) are projected to become the second leading cause of cancer-related deaths by 2030 [1]. The survival curve is extremely short (6 months) and the survival rate at 5 years is very low (3%). This dramatic outcome is related to a lack of therapeutic tools and early diagnostic markers which makes pancreatic cancer the most deadly cancer. At the time of diagnosis, more than 80% of PC are already metastatic or locally advanced and only about 10% to 15% of patients are considered eligible for surgical resection [2]. Remaining patients that do not benefit from surgery will receive palliative chemotherapy and notably gemcitabine, a fluorinated analog of deoxycytidine that is a major chemotherapeutic drug used in firstline in advanced PC. Unfortunately, PC is characterized by both an intrinsic and an acquired chemoresistance associated with different mechanisms that lead to relapse and death [3,4]. Deciphering mechanisms responsible for PC cell resistance to gemcitabine is thus crucial to improve efficacy of the drug and propose more efficient therapies.

A better understanding of the signalling pathways and complex gene networks that are altered during carcinogenesis progression may help design new therapeutic strategies. Among alterations occurring in pancreatic cancer, TGF-β signalling pathway is frequently lost as SMAD4/DPC4 (deleted in pancreatic cancer 4) is mutated in 50–80% of PDAC and mutations of TGF-βRII are also described (5–10%) [3,5]. Interestingly, TGF-β initially inhibits epithelial growth whereas it appears to promote the progression of advanced tumors and thus emerged as tumor suppressor pathway in pancreatic cancer [6]. TGF-β can act in an autocrine manner or as a paracrine factor secreted by the microenvironment [7]. After binding to its receptor TGF-βRII, TGF-β signals via activation of several pathways. The canonical pathway involves the Smad proteins, but activation of other pathways such as MAPKs, PI3K or small GTPases [7] pathways may also mediate TGF-β effects. We previously showed that TGF-β can regulate MUC4 expression via canonical or alternative signalling pathways [8] and that MUC4 is involved in gemcitabine resistance in PC cells [9].

Therefore, to better understand the role and contribution of TGF-βRII in TGF-β signalling and biological properties of PC cells in vitro and in vivo, we developed two PC cell lines stably knocked down for TGF-βRII. Our results show that TGF-βRII silencing promotes tumor growth but reduces migration and increases resistance to gemcitabine in vitro and in vivo. TGF-βRII silencing also leads to STAT3 and c-Jun phosphorylation, alteration of MRP3 and MRP4 ABC transporters expression and induction of a partial EMT phenotype.

This work underlies the importance of TGF-β signaling pathways and associated cellular mechanisms as inducers of chemoresistance to gemcitabine and proposes potential new therapeutic tools to clinicians, surgeons and anatomopathologists for this deadly disease.

## 2. Results

### 2.1. Generation and Characterization of Stable TGF-βRII-KD Cell Lines

**Figure 1:**
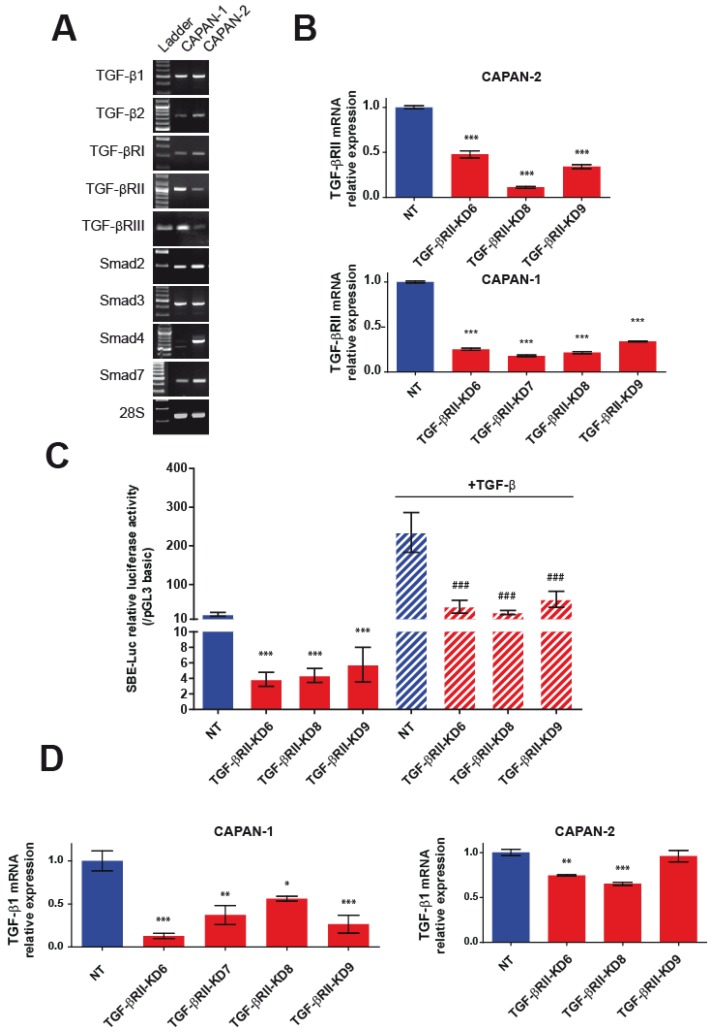
Characterization of TGF-β/Smad pathway in TGF-βRII-KD CAPAN-1 and CAPAN-2 cell lines. (**A**) Analysis of mRNA expression of TGF-β1, TGF-β2, TGF-βRI, TGF-βRII, TGF-βRIII, Smad2, Smad3, SMAD4, Smad7 and 28S in CAPAN-1, CAPAN-2 cells by RT–PCR. (**B**) Analysis of mRNA relative expression of TGF-βRII in NT and TGF-βRII-KD CAPAN-1 and CAPAN-2 cell lines. Expression in NT cells was arbitrarily set to 1. (**C**) Smad-Binding-Elements (SBE)-Luc relative luciferase activity in untreated and TGF-β treated NT and TGF-βRII-KD CAPAN-2 cells. Relative luciferase activity was expressed as a ratio of SBE-Luc normalized with pGL3 basic activity. (**D**) Analysis of mRNA relative expression of TGF-β1 in NT and TGF-βRII-KD CAPAN-1 and CAPAN-2 cell lines. N = 3. * *p* < 0.05, ** *p* < 0.01 and *** *p* < 0.001 indicate statistical significance compared with the NT control. ### *p* < 0.001 indicate statistical significance compared with the TGF-β treated NT control.

Expression of TGF-βRI, TGF-βRII, TGF-βRIII, Smad2, Smad3, and Smad7 was confirmed by RT-PCR in CAPAN-1 and CAPAN-2 cells. Wild type SMAD4, as it is mutated, is not detected in CAPAN-1 [8,10]. Altogether this suggests that CAPAN-2 cells harbor a functional TGF-β signaling pathway whereas the canonical Smad pathway is not functional in CAPAN-1 cells. Moreover, strong TGF-β1 and mild TGF-β2 mRNA levels were observed in both cell lines suggesting TGF-β growth factor autocrine expression (Figure 1A).

We generated CAPAN-1 and CAPAN-2 stable cell lines in which TGF-βRII was knocked down (TGF-βRII-KD) by a shRNA approach. Four different shRNA sequences were used to establish four different cell lines designated as TGF-βRIIKD6, TGF-βRIIKD7, TGF-βRIIKD8 and TGF-βRIIKD9. Using qPCR, we confirmed that TGF-βRII mRNA levels are decreased in all CAPAN-1 and CAPAN-2 TGF-βRII-KD cells compared to NT control cells (*p* < 0.005, ***) (Figure 1B). We were not able to produce TGF-βRIIKD7 cell line in CAPAN-2.

In CAPAN-2 KD cells, the inhibition of TGF-βRII expression was correlated with a loss of activity of the Smad binding elements (SBE)-Luc synthetic promoter (Figure 1C). In CAPAN-2 NT cells, TGF-β treatment induces a 10-fold increase of SBE-Luc relative activity whereas we observed a lesser SBE-Luc activity in TGF-βRII-KD cells (*p* < 0.001). As expected, in CAPAN-1 cells mutated for SMAD4, we did not observe any activity of SBE-Luc construct with or without TGF-β treatment (not shown). Interestingly, TGF-βRII knocking down led to decreased TGF-β1 mRNA level in CAPAN-1 TGF-βRII-KD cells (44–87% decrease) (Figure 1D) whereas the effect was less pronounced (21–25%) in TGF-βRII-KD CAPAN-2 cell lines.

### 2.2. Involvement of TGF-βRII in PC Cell Biological Properties

**Figure 2:**
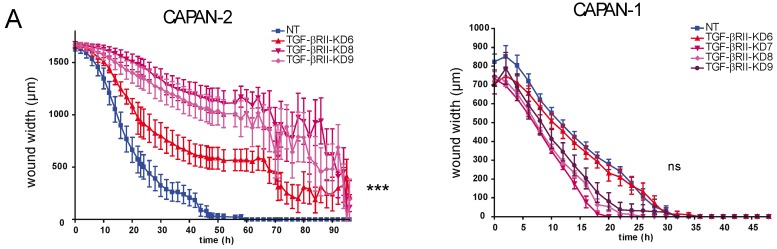

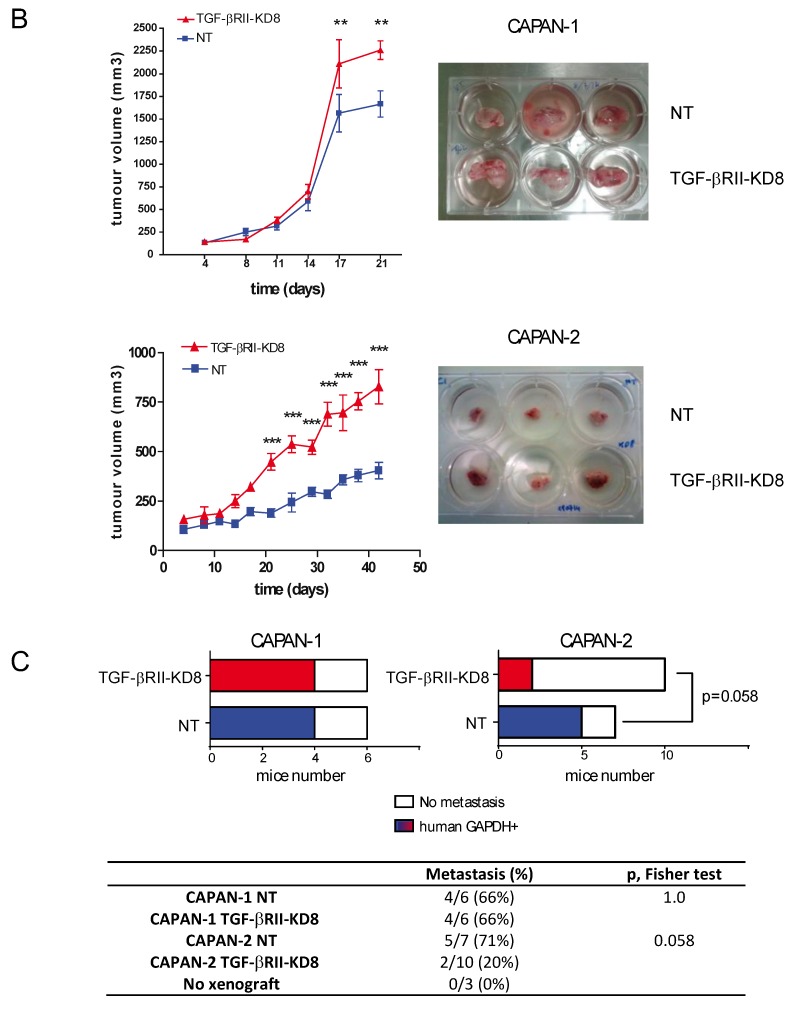
TGF-βRII alters tumor growth and migration in pancreatic cancer cells. (**A**) Wound healing closure of NT and TGF-βRII-KD CAPAN-1 and CAPAN-2 cell lines using the IncuCyte™ chamber apparatus. N = 3. (**B**) Subcutaneous xenografts of NT/TGF-βRII-KD8 CAPAN-1 and CAPAN-2 cells in *SCID* mice. Tumour growth (mm3) was evaluated until sacrifice. ** *p* < 0.01 and *** *p* < 0.001 indicate statistical significance of TGF-βRII-KD compared with the NT control. ns: not significant. (**C**) Evaluation of the presence of micro-metastases in the liver by detecting the presence of human GAPDH in the liver of xenografted mice (NT and TGF-βRII-KD CAPAN-1 and CAPAN-2) by qPCR.

We investigated the effect of TGF-βRII silencing on CAPAN-1 and CAPAN-2 proliferation and migration properties. Cell migration was assessed by wound healing test. In CAPAN-2 NT cells, the wound was entirely closed at 60 h. In CAPAN-2 TGF-βRII-KD cells, we observed a strong delay of wound closure that was statistically significant at 16–18 h (*p* < 0.001, ***) (Figure 2A, left panel). Interestingly, we did not observe any statistically significant difference in wound closure in CAPAN-1 TGF-β-RIIKD or NT cells suggesting the involvement of a functional SMAD4 signaling pathway in wound closure (Figure 2A, right panel). TGF-βRII-KD CAPAN-1 or CAPAN-2 cells also showed a trend toward increased proliferation at 96 h compared to the respective NT control cells but that remained not significant (not shown).

In order to determine the role of TGF-βRII on pancreatic carcinogenesis in vivo, CAPAN-1/-2 TGF-βRII-KD8 and NT SC xenograft studies were carried out. We selected the TGF-βRII-KD8 cell lines for in vivo studies as this cell line harboured the best KD in CAPAN-1 and CAPAN-2. The results indicate that the tumour volume was significantly higher in xenografted mice with CAPAN-1 TGF-βRII-KD8 compared to CAPAN-1 NT controls. The relative tumour volume was 2.26 ± 0.1 cm^3^ when compared to NT control tumour volume (1.66 ± 0.14 cm^3^) at day 21. The increase was statistically significant (**, *p* < 0.01). Similar results were obtained with CAPAN-2 TGF-βRII-KD8 xenografts (0.423 ± 0.05 vs. 0.828 ± 0.08 cm^3^) at day 42 (Figure 2B). Furthermore, we also evaluated the presence of micro-metastasis in the liver by detecting the presence of human GAPDH in the liver of the mouse by qPCR (Figure 2C). We detected micro-metastases in 5/7 (71%) CAPAN-2 controls whereas only 2/10 (20%) of CAPAN-2 TGF-βRII-KD8 xenografted mice harboured micro-metastases. Contingency analysis showed that difference was close to statistical significance (*p* = 0.058). We did not observe any difference in CAPAN-1 TGF-βRII-KD8 (4/6) compared to CAPAN-1 NT controls (4/6). No human GAPDH mRNA was detected in mice without xenografts. Our results suggest that TGF-βRII signalling is involved in tumor growth and migration of pancreatic cancer cells both in vitro and in vivo.

### 2.3. Role of TGF-βRII on PC Cells Sensitivity to Gemcitabine

**Figure 3:**
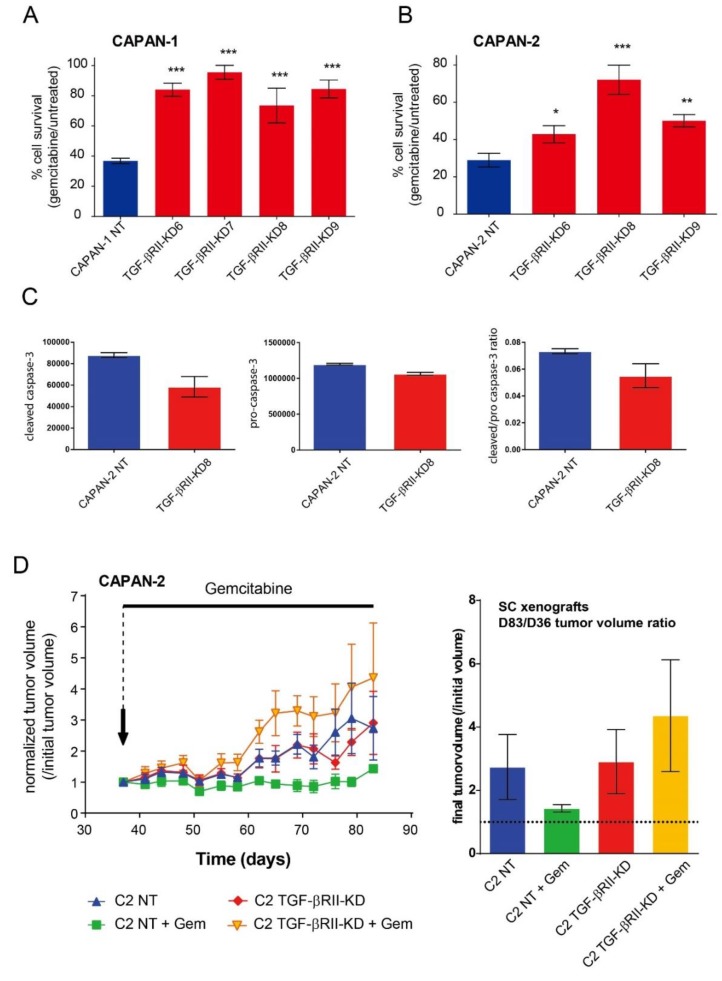
TGF-βRII alters sensitivity to gemcitabine in pancreatic cancer cells in vitro and in vivo. Survival rates in different TGF-βRII-KD CAPAN-1 (**A**) and CAPAN-2 (**B**) cell lines or their NT control cells were measured following treatment with gemcitabine using the MTT assay. Results are expressed as % of cell survival (/untreated cells). Three independent experiments were performed. (**C**) Pro and cleaved caspase-3 relative expression were analysed using Human Apoptosis Array Kit in TGF-βRII-KD CAPAN-2 cells following gemcitabine treatment. Cleaved/pro-caspase-3 ratio was calculated (**D**) Subcutaneous xenografts of NT and TGF-βRII-KD8 CAPAN-2 cells in *SCID* mice. Gemcitabine (15 mg/kg) or PBS (200 µL) were injected intra-peritoneously, twice a week once palpable tumors were developed. Normalized tumor growth is expressed as the ratio of tumor progression relative to tumor volume on the first day of gemcitabine treatment. Right graph represents tumor growth over time. Left graph represents final tumor volume at day 83 (normalized as initial tumor volume at D36 equal to 1). * *p* < 0.05, ** *p* < 0.01 and *** *p* < 0.001 indicate statistical significance compared with the NT control.

We investigated the effect of TGF-βRII silencing on CAPAN-1 and CAPAN-2 cell sensitivity to gemcitabine. We show that the lack of TGF-βRII induces a significant increase of resistance to gemcitabine treatment in both CAPAN-1 (87–152% increase of survival rate, Figure 3A) and CAPAN-2 (50–161% increase, Figure 3B) cell lines compared to NT control cells. All differences were statistically significant. Pro and cleaved caspase-3 relative expression were analysed using Human Apoptosis Array Kit in TGF-βRII-KD CAPAN-2 cells following gemcitabine treatment. We showed a decrease of cleavage of caspase3 in TGF-βRII-KD cells compared to NT cells (Figure 3C). We then carried out SC xenograft of NT or TGF-βRII-KD8 CAPAN-2 cells that were subsequently treated with gemcitabine for 46 days. Gemcitabine treatment stabilized the normalized tumor volume in CAPAN-2 NT xenografts (2.7 ± 1.02 vs. 1.4 ± 0.11 at D83) compared to initial tumor volume (D36). On the contrary, the tumor growth was exacerbated in TGF-βRII-KD xenografts following gemcitabine treatment (2.9 ± 1.7 vs. 4.36 ± 1 at D83) (Figure 3D). Altogether, our results suggest that TGF-βRII alters sensitivity of PC cells to gemcitabine both in vitro and in vivo.

### 2.4. Identification of Signalling Pathways Altered following TGF-βRII Knocking Down

**Figure 4:**
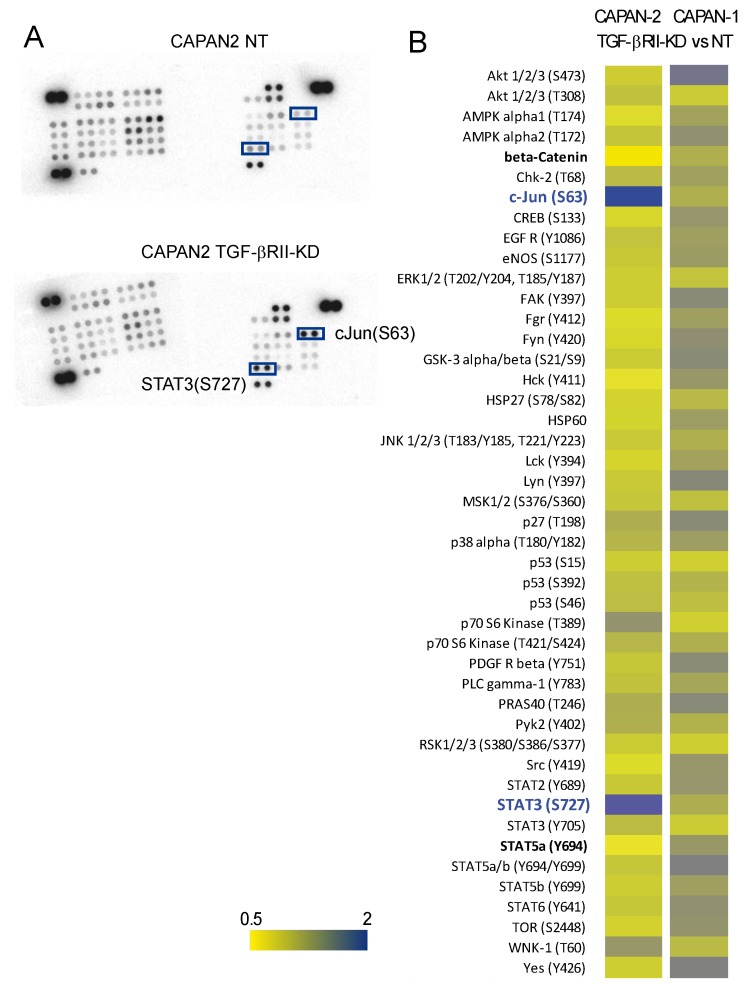
Impact of TGF-βRII knocking-down on signaling pathways. (**A**) Impact of TGF-βRII knocking-down on intracellular signaling was studied using phospho-arrays that detect relative site-specific phosphorylation of 43 proteins. Boxes highlight spots for S63 c-Jun and S727 STAT3. (**B**) Heatmap representing the intensities of each spot (TGF-βRII vs. NT) that were measured and normalized to the reference spots for CAPAN-1 and CAPAN-2 cells.

**Figure 5:**
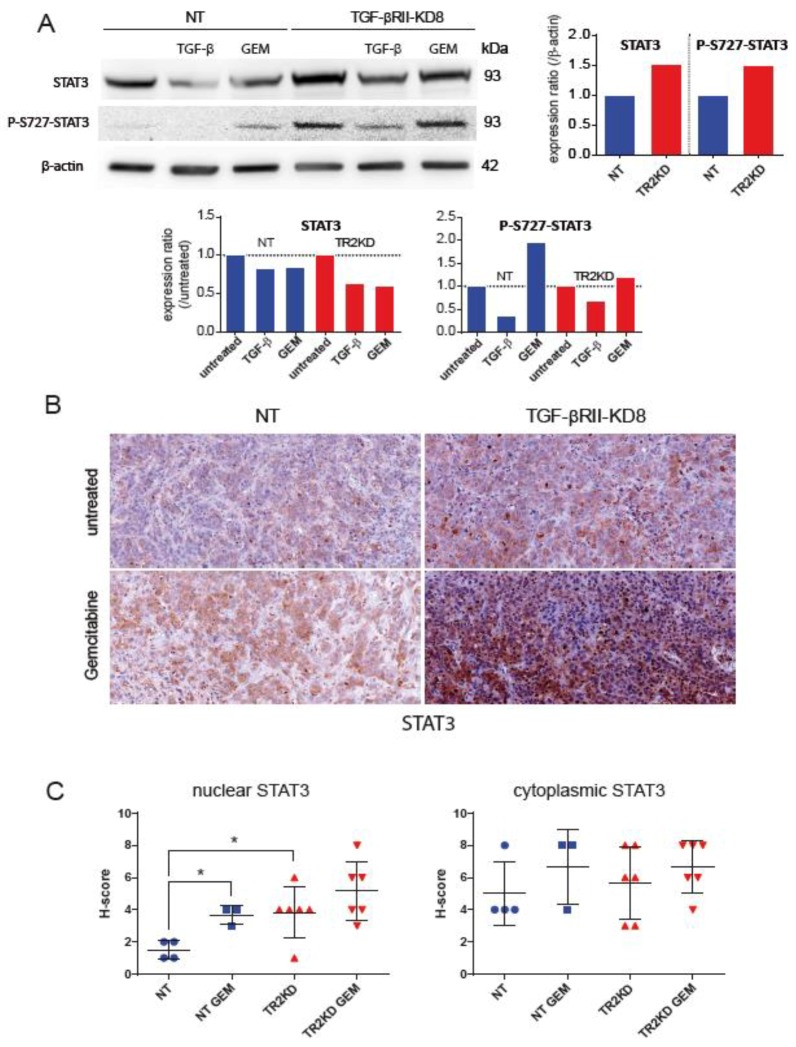
TGF-βRII knockdown promotes STAT3 phosphorylation and nuclear localization in CAPAN-2 cells. (**A**) STAT3, phospho-S727 STAT3 and β-actin expression was analysed by western blotting. Bands intensities were quantified by densitometry and ratios (KD vs. NT or treated/untreated) are indicated in the graphs. Expression in NT (for TGF-βRIIKD) or untreated (for gemcitabine/TGF-β) cells was arbitrarily set to 1. (**B**) IHC analysis of STAT3 on extracted xenografted NT and TGF-βRIIKD tumors. (**C**) Nuclear and cytoplasmic IHC staining were scored in NT and TGF-βRIIKD xenografted tumors that were treated with gemcitabine or PBS. * *p* < 0.05 indicates statistical significance of TGF-βRII-KD1 compared with the NT control.

Impact of TGF-βRII knocking-down on intracellular signaling was studied using phospho arrays that detect relative site-specific phosphorylation of 43 proteins simultaneously (Figure 4). Intensities of each spot for TGF-βRII were measured and normalized to the CAPAN-2 NT proteins (Figure 4A). We observed an important increase of phosphorylation of S63 c-Jun (3.3-fold) and S727 STAT3 (1.5-fold) in CAPAN-2 TGF-βRII-KD8 compared to NT cells. We also observed a mild decrease of phosphorylation of Y694 STAT5a (0.6-fold) and β-catenin (0.5-fold) (Figure 4B). Similar experiments were conducted for CAPAN-1 TGF-βRII-KD8 and NT cells. Only weak variations were observed (<30%). By western blotting, we confirmed the increased of phospho-S727 STAT3 (4.49-fold) (Figure 5A) and phospho-S63 c-Jun (7-fold) (Supplementary Figure S1A) in CAPAN-2 TGF-βRII-KD8 cells compared to CAPAN-2 NT cells.

Gemcitabine treatment also induced an increase of phospho-S727 STAT3 (1.95-fold) (Figure 5A) and phospho-S63 c-Jun (3.18-fold) (Supplementary Figure S1A) in NT cells (compared to untreated cells). This effect was not found in TGF-βRII-KD8 cells. We then performed immunohistochemistry for STAT3 and c-Jun in NT or TGF-βRII-KD8 CAPAN-2 SC xenografts (Figure 5B and Supplementary Figure S1B). Nuclear and cytoplasmic IHC staining were scored. We show that STAT3 nuclear H-score in TGF-βRII-KD8 tumors was significantly higher than in NT tumors (*, *p* = 0.0429) (Figure 5C). We also observed that STAT3 nuclear staining was increased following gemcitabine treatment (*, *p* = 0.0286). 

A mild increase of nuclear STAT3 was observed in TGF-βRII-KD8 tumors following gemcitabine treatment but was not statistically significant (*p* = 0.33) (Figure 5C). No alteration of c-jun expression was observed in NT and TGF-βRII-KD8 untreated xenograft tumors (Supplementary Figure S1B). H score measurement indicates that gemcitabine treatment led to a significant decrease of c-Jun staining in TGF-βRII-KD8 tumors (Supplementary Figure S1C). Altogether, our results indicate that TGF-βRII signalling implicates STAT3 and c-Jun phosphorylation in pancreatic cancer cells.

### 2.5. TGF-βRII Silencing Alters the Expression of ABC Transporters and EMT Markers in PC Cells

**Figure 6:**
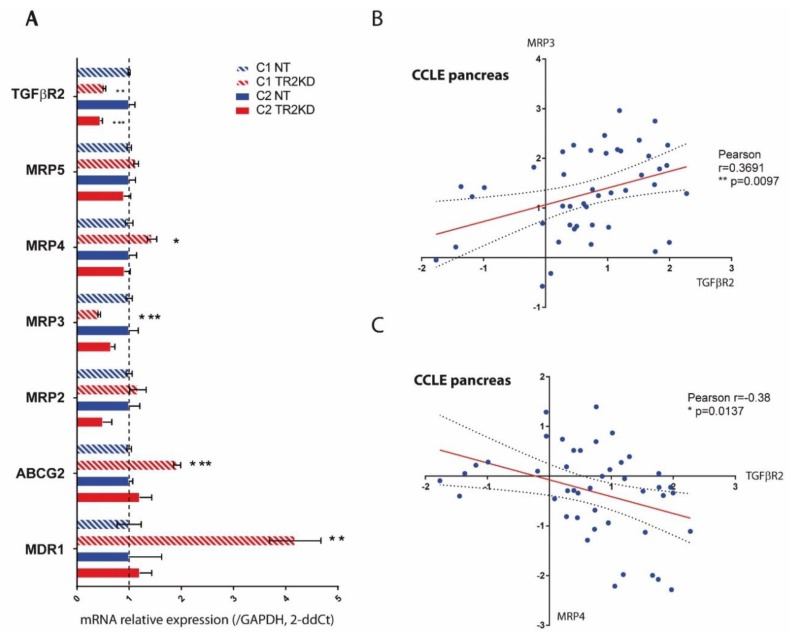
TGF-βRII silencing alters ABC transporter expression. (**A**) mRNA expression of *TGF-βRII*, *MRP1*, *MRP2*, *MRP3*, *MRP4*, *MRP5*, *ABCG2* and *MDR1* was analyzed in NT and TGF-βRII-KD CAPAN-1 and CAPAN-2 cells by qRT–PCR. The histogram represents the ratio of their expression in TGF-βRII-KD compared with NT cells. Three independent experiments were performed. * *p* < 0.05, ** *p* < 0.01 and *** *p* < 0.001 indicate statistical significance of TGF-βRII-KD1 compared with the NT control. TGF-βRII, MRP3 (**B**) and MRP4 (**C**) mRNA expression was extracted from PC cell lines from the Cancer Cell Line Encyclopedia (CCLE). Statistical analyses of MRP3/TGF-βRII and MRP4/TGF-βRII correlations were performed using Pearson’s correlation coefficient.

To go further and understand which molecular mechanisms could be responsible for the induced chemoresistance, we investigated the effect of TGF-βRII silencing on the expression of ATP-binding cassette (ABC) transporters that are commonly known to confer resistance to xenobiotics including chemotherapeutic drugs. Using qPCR, we investigated the expression of *ABCB1/MDR1*, *ABCC1/MRP1*, *ABCC2/MRP2*, *ABCC3/MRP3*, *ABCC4/MRP4*, *ABCC5/MRP5* and *ABCG2* in NT and TGF-βRII-KD CAPAN-1 and CAPAN-2 cells. *MRP1* was not detected. We found that *MDR1* (×4.2-fold, **), *ABCG2* (×1.9-fold, ***) and *MRP4* (×1.4-fold, *) mRNA levels were significantly increased in TGF-βRII-KD CAPAN-1 cells compared to NT cells (Figure 6A). 

*MRP3* mRNA level was decreased in TGF-βRII-KD CAPAN-1 cells (×0.42-fold, ***) and CAPAN-2 (×0.65-fold, *p* = 0.13) (Figure 6A). TGF-βRII and ABC transporter expression was analyzed from 44 pancreatic cancer cell lines from CCLE. We showed that TGF-βRII mRNA relative level was correlated with expression of MRP3 (Pearson r = 0.3856, *p* = 0.0097) (Figure 6B) and conversely correlated with MRP4 (Pearson r = −0.3691, *p* = 0.037) (Figure 6C).

Furthermore, TGF-β is commonly described as an inducer of epithelial-mesenchymal transition (EMT) that is associated with chemoresistance [11]. We performed vimentin (mesenchymal marker) and E-cadherin (epithelial marker) immunohistochemical staining on FFPE sections of NT or TGF-βRII-KD CAPAN-2 xenografts treated with gemcitabine to check their status. Surprisingly, we observed a slight increase of vimentin in TGF-βRII-KD CAPAN-2 cells xenografts compared to NT tumors (Supplementary Figure S2). Moreover, we found that gemcitabine treatment induced a loss of E-cadherin staining and a gain of vimentin staining suggesting an EMT transdifferantiation process following gemcitabine treatment. As vimentin staining is not homogenous, we cannot exclude that vimentin is not expressed by activated pancreatic stellate cells within the tumor.

Altogether, these results suggest that TGF-βRII silencing alters expression of MRP3 and MRP4 ABC transporters in PC cells and induces a partial EMT phenotype that could promote chemoresistance to gemcitabine.

## 3. Discussion

In the present manuscript, we showed that TGF-βRII silencing promotes tumor growth but also reduces migration and increases resistance to gemcitabine in vitro and in vivo. TGF-βRII silencing also leads to STAT3 and c-Jun phosphorylation, alteration of MRP3 and MRP4 ABC transporters expression and induction of a partial EMT phenotype.

TGF-β signalling pathway has been described as a double edge sword during carcinogenesis [12]; acting as a tumor suppressor in the early stages but promoting metastasis in the advanced carcinoma [6]. Moses’s laboratory generated TGF-βRII knock out mice crossed with Ptf1a-Cre; LSL-KrasG12D and showed that compound mice developed well differentiated PDAC [13] mostly highlighting the role as a tumor suppressor. TGF-βRII targeting by a monoclonal antibody is also effective at reducing metastasis [14]. In our cellular models, we confirmed that TGF-βRII inhibition led to an increased tumor growth in vivo and TGF-βRIIKD CAPAN-2 tumors led to less metastasis in the liver. We hypothesize that therapeutic silencing of TGF-βRII lead to reduced metastasis burden. For example, such strategies targeting TGF-βRII expression are developed using post-transcriptional modulators such as microRNA in colorectal cancer [15,16]. Potential use in pancreatic adenocarcinoma remains to be proven. However, as TGF-βRII silencing also promotes chemoresistance, we propose that targeting downstream mediators of TGF-βRII could be an interesting alternative. STAT3 targeting has been proposed as a therapeutic option in pancreatic adenocarcinoma [17]. Combined treatments of gemcitabine and a JAK inhibitor (AZD1480) led to stroma remodeling, increased density of microvessel, enhanced drug delivery and improved survival of in Ptf1a-Cre; LSL-KrasG12D; TGF-βRII^KO^ vivo models suggesting an effect of the treatment via the stroma [18]. STAT3 S727 phosphorylation was previously studied in prostate carcinogenesis and was shown to promote cell survival and cell invasion [19]. In the present work, we also showed that TGF-βRII inhibition led to STAT3 S727 phosphorylation and increased gemcitabine resistance of the tumor cells suggesting the crucial role of STAT3 in both tumor and stromal cells. STAT3 knockdown was shown to be associated with increased response to gemcitabine in pancreatic cancer cells [20]. It is interesting to note that erlotinib treatment that inhibits epidermal growth factor receptor (EGFR) tyrosine kinase also inhibited phosphorylation of STAT3 [21]. Among the targeted therapy for PDAC, erlotinib associated with gemcitabine is the only drug showing statistically significantly improved survival [22].

TGF-β signaling is mediated through canonical SMAD and non-canonical non-SMAD pathways [6]. Accordingly, we only observed increase of c-Jun and STAT3 phosphorylation in CAPAN-2 TGF-βRIIKD cells but not in the CAPAN-1 model that is SMAD4/p53/BRCA2 mutated [23,24]. It was previously shown that STAT3-induced senescence requires functional TGFβR signaling and notably a functional SMAD3/SMAD4 pathway. STAT3 promotes SMAD3 nuclear localization [25]. We hypothesize that the TGF-βRIIKD-induced gemcitabine resistance, shown in the present manuscript, is mediated by STAT3 which similarly requires SMAD3/SMAD4 dependent pathway.

In SMAD4 mutated CAPAN-1 cells we observed an increased expression of MRP4, ABCG2 and MDR1. This increased expression could be responsible for the gemcitabine resistance of CAPAN-1 TGF-βRIIKD cells. The link between ABC transporters and TGF-β pathway is scarcely described. TGF-β1 has been shown to upregulate ABCG2 expression in MiaPACA2 PC cells which is contradictory with our findings [26]. In breast cancer cells, silencing of TGF-βRII leads to overexpression of multidrug resistance protein ABCG2 and tamoxifen resistance [27].

TGF-β is usually considered as a bona fide inducer of EMT [28]. However, we were surprised to observe that TGF-βRII inhibition led to a partial EMT with an increase of vimentin. STAT3 signaling is linked to cancer cell plasticity and is able to promote EMT and CSC expansion [29]. Previous work also showed that IL6, secreted by pancreatic stellate cells, triggers STAT3 activation in pancreatic cells which subsequently induces EMT via Nrf2 [30]. Therefore, we hypothesize that the paradoxal EMT observed in TGF-βRII cells is a consequence of the STAT3 phosphorylation on S727.

## 4. Materials and Methods

### 4.1. Cell Culture

CAPAN-1 and CAPAN-2 PC cell lines were cultured as previously described [8]. CAPAN-1 and CAPAN-2 are both KrasG12V mutated. CAPAN-1 cells express a truncated BRCA2 protein and harbor inactivating mutation in p53 and SMAD4. CAPAN-2 cells express wildtype p53 and normal levels of SMAD4 protein [23,24]. TGF-βRII-knocked down (KD) cells were obtained following stable transfection of CAPAN-1 and CAPAN-2 cells with four different pGeneClipTM puromycin vectors encoding TGF-βRII ShRNA (SA Biosciences^TM^, Venlo, The Netherlands) as previously described [31]. The empty vector was used to raise control clones called Non Targeting (NT). Four selected clones of NT and each TGF-βRII-KD cells were pooled in order to avoid clonal variation and were designated TGF-βRIIKD6, TGF-βRIIKD7, TGF-βRIIKD8 and TGF-βRIIKD9. All cells were maintained in a 37 °C incubator with 5% CO_2_ and cultured as the parental cells.

### 4.2. qRT-PCR

Total RNA from PC cells was prepared using the NucleoSpin^®^ RNA II kit (Macherey Nagel, Hoerdt, Germany). cDNA was prepared as previously described [32]. Semi-quantitative PCR was performed as previously described [33]. qPCR was performed using SsoFastTM Evagreen Supermix kit following the manufacturer’s protocol using the CFX96 real time PCR system (Bio-Rad, Hercules, CA, USA). Primer information is given in Table 1. Each marker was assayed in triplicate in three independent experiments. Expression level of genes of interest was normalized to the mRNA level of GAPDH housekeeping gene.

### 4.3. Protein Extraction and Western-Blotting

Total cellular extracts were performed as previously described in Van Seuningen et al. [34] and Jonckheere et al. [35]. Western-blotting on nitrocellulose membrane (0.2 µm, Whatman, Maidstone, UK) was carried out as previously described [36]. Membranes were incubated with antibodies against STAT3 (79D7, Cell signalling), phospho S727 STAT3 (9134, signalling), c-Jun (60A8, Cell signalling), phospho S63 c-Jun (54B3, Cell signalling) and β-actin (AC-15, Sigma, Tokyo, Japan). Antibodies were diluted in 5% (*w*/*v*) non-fat dry milk in Tris-Buffered Saline Tween-20 (TBS-T). Peroxydase-conjugated secondary antibodies (Sigma-Aldrich, St. Louis, MO, USA) were used and immunoreactive bands were visualised using the West Pico chemoluminescent substrate (Thermo Scientific, Pierce, Brebières, France). Intracellular signaling was studied using Human Phospho-Kinase Antibody Array (ARY003B, R & D) (detecting relative site-specific phosphorylation of 43 proteins) and Human Apoptosis Array Kit (ARY009, R & D). Chemo-luminescence was visualised using LAS4000 apparatus (Fujifilm, Tokyo, Japan). Density of bands were integrated using Gel analyst software^®^ (Claravision, Paris, France) and represented as histograms. Three independent experiments were performed.

### 4.4. Cell Proliferation

Cells were seeded at 1 × 10^5^ cells per well in 6-well plates. Cells were counted daily using a Malassez counting chamber using Trypan Blue exclusion dye (Life Technologies, Carlsbad, CA, USA) during 96 h. Experiments were performed three times in triplicate.

### 4.5. Wound Healing Test

1500 cells were seeded per wells in 96 well plates (Image LockTM plates, Essen Bioscience, Ann Arbor, MI, USA) and cultured until confluence was reached. The wound was realized using IncuCyte wound maker (Essen BioScience). Cells were washed three times with PBS 1X and complete medium was added to the cells. Wound widths were analyzed using Incucyte platform (Live-Cell imaging System, Essen Bioscience) and pictures collected every 2 h.

### 4.6. Cytotoxicity Assay

Cells were seeded in growth medium into 96-well plates at a density of 10^4^ cells per well. After 24 h incubation, the medium was replaced by fresh medium containing gemcitabine at 35 nM and incubated for 72 h at 37 °C. The viability of cells was determined using the 3-(4,5-dimethylthiazol-2-yl)-2,5-diphenyltetrazolium bromide assay (MTT, Sigma-Aldrich) as previously described [9]. Percentage of viability = [(A_treated_ − A_blank_)/(A_neg_. − A_blank_)] × 100; where A_treated_ is the average of absorbance in wells containing cells treated with gemcitabine, A_neg_. is the average of wells containing cells without gemcitabine treatment, and A_blank_ is the average of wells containing medium without cells.

### 4.7. Subcutaneous Xenografts

NT or TGF-βRII-KD CAPAN-1 (10^6^ cells in 100 μL Matrigel) and CAPAN-2 (2 × 10^6^) cells were injected subcutaneously (SC) into the flank of seven-week-old male Severe Combined Immunodeficient (*SCID*) mice (CB17, Janvier, France). Six mice were used per group. Tumor size was evaluated weekly by measuring the length (L) and the width (W) and tumor volume was calculated with the formula (W^2^ × L). Once palpable tumors were developed (250 mm^3^), gemcitabine (15 mg/kg) or PBS (200 µL) were injected intra-peritoneously, twice a week. All procedures were in accordance with the guideline of animal care committee (Comité Ethique Expérimentation Animale Nord Pas-de-Calais, Lille, France, #122012).

### 4.8. Immunohistochemistry

Subcutaneaous NT/TGF-βRII-KD CAPAN-1/CAPAN-2 xenografts were fixed in 4% (*w*/*v*) buffered formaldehyde, embedded in paraffin, cut at 4 µm thickness and applied on SuperFrost^®^ slides (Menzel-Glaser, Braunschweig, Germany). Manual IHC was carried out as previously described [37]. Briefly, slides were deparaffinised using a series of xylol–ethanol baths. Endogenous peroxidase activity was inactivated by H_2_O_2_ (1.5%, *v*/*v*, 30 min) followed by antigen retrieval Dako Real citrate buffer (microwave 700 W, 20 min). Thereafter, sections were incubated with TENG-T (10 nM Tris–HCl pH 8 containing 5 mM EDTA, 150 mM NaCl, 0.25% (*w*/*v*) gelatin, and 0.05% (*w*/*v*) Tween 20) for 30 min to reduce nonspecific binding. The antibodies were used as followed: anti-STAT3 (1:200, #483 Santa Cruz), anti-c-Jun (1:200, 60A8 Cell signaling), anti-E-Cadherin (1:200, 3195 Cell signalling) and anti-vimentin (1:200, sc5741, Santa Cruz). Sections were incubated for 1 h with biotinylated rabbit IgG (Vector Laboratories, Peterborough, UK) followed by a 1 h incubation with ABC/PO complex (Vectastain Elite Kit, Vector Laboratories). Intensity of staining was graded as weak (1), moderate (2) or strong (3). The percentage of ductal stained cells was graded as 1 (0–25%), 2 (25–50%), 3 (50–75%) and 4 (75–100%). Total score was calculated by multiplying the intensity score and percentage score.

### 4.9. Expression Analysis in CCLE Database

TGF-βRII, ABCB1/MDR1, ABCC1/2/3/4/5 and ABCG2 z-score expressions were extracted from databases available at cBioPortal for Cancer Genomics [38,39]. The queries were realized in CCLE (44 pancreatic samples, Broad Institute, Novartis Institutes for Biomedical Research, Cambridge, MA, USA) [40].

### 4.10. Statistical Analyses

Statistical analyses were performed using the Graphpad Prism 6.0 software (Graphpad Software Inc., La Jolla, CA, USA). Differences in data of two samples were analysed by the student’s *t* test or ANOVA test with selected comparison using tukey post-hoc test and were considered significant for *p*-values < 0.05 *, *p* < 0.01 ** or *p* < 0.001 ***/###.

## 5. Conclusions

In the present manuscript, we characterized CAPAN-1 and CAPAN-2 pancreatic cancer cell lines stably invalidated for TGF-βRII and investigated the consequences on both their biological properties and response to gemcitabine in vitro and in vivo. We show an increase of tumor growth and a reduction of cell migration. We also show for the first time an increased resistance to gemcitabine that could be mediated by S727 STAT3 phosphorylation and via deregulation of MRP3 and MRP4 ABC transporter expression.

This work underlies the importance of TGF-β signaling pathways and associated cellular mechanisms, such as c-Jun, STAT3 or MRP ABC transporters as inducers of chemoresistance to gemcitabine and proposes potential new therapeutic options to better treat/manage this deadly disease.

## Figures and Tables

**Table 1:** Primers used for RT-PCR and qPCR experiments.

Gene	Orientation	Sequences of Primers (5′–3′)	T°m (°C)	Expected Size
RT-PCR				
*28S*	Forward Reverse	GCAGGGCGAAGCAGAAGGAAACT TGAGATCGTTTCGGCCCCAA	59	231
*TGF-β1*	Forward Reverse	GAGGTGACCTGGCCACCATTCAT CCAGCCGAGGTCCTTGCGGA	60	194
*TGF-β2*	Forward Reverse	GCTTTTCTGATCCTGCATCTG CAATACCTGCAAATCTTGCTTC	56	823
*TGF-βRI*	Forward Reverse	CTCTCCTTTTTTCTTCAGATCTGC AATCCAACTCCTTTGCCCTT	55	328
*TGF-βRII*	Forward Reverse	GCCAACAACATCAACCACAACACA TAGTGTTTAGGGAGCCGTCTTCAG	61	1003
*TGF-βRIII*	Forward Reverse	TGCCTTACTTCTCTTGCCTTAA GCAAAGTGGCATCATATTATT	56	100
*Smad2*	Forward Reverse	GTCCATCTTGCCATTCACG TGGTGATGGCTTTCTCAAGC	55	192
*Smad3*	Forward Reverse	GGGCTCCCTCATGTCATCTA GGCTCGCAGTAGGTAACTGG	60	443
*SMAD4*	Forward Reverse	CTCCTGAGTATTGGTGTTCC CTAAAGGTTGTGGGTCTGC	56	796
*Smad7*	Forward Reverse	GGCTCGCAGTAGGTAACTGG TTGTTGTCCGAATTGAGCTG	55	448
qPCR				
*TGF-β1*	Forward Reverse	CACTCTCAAACCTTTACGAGACC CGTTGCTAGGGGCGAAGATG	58	131
*TGF-βRII*	Forward Reverse	AGGAGTATGCCTCTTGGAAGAC AGCCAGTATTGTTTCCCCAAC	58	123
*Human GADPH*	Forward Reverse	CCACATCGCTCAGACACCAT CCAGGCGCCCAATACG	58	70
*Mouse GADPH*	Forward Reverse	AGGTCGGTGTGAACGGATTTG TGTAGACCATGTAGTTGAGGTCA	58	129

## Supplementary Materials

The following are available online at http://www.mdpi.com/2072-6694/10/8/254/s1. Supplementary Figure S1, TGF-βRII knockdown promotes c-Jun S-63 phosphorylation in CAPAN-2 cells. (A) c-Jun, phospho-S63 c-Jun and β-actin expression was analysed by western blotting. Bands intensities were quantified by densitometry and ratios (KD vs. NT or treated/untreated) are indicated in the graphs. Expression in NT (for TGF-βRIIKD) or untreated (for gemcitabine/TGF-β) cells was arbitrarily set to 1. (B) IHC analysis of c-Jun on extracted xenografted NT and TGF-βRIIKD tumors. (C) IHC staining was scored in NT and TGF-βRIIKD xenografted tumors that were treated with gemcitabine or PBS. * *p* < 0.05 indicate statistical significance of TGF-βRII-KD compared with the NT control. Supplementary Figure S2, TGF-βRII knockdown promotes partial EMT-like phenotype. IHC analysis of E-cadherin and vimentin on extracted xenografted NT and TGF-βRIIKD tumors. IHC staining was scored in NT and TGF-βRIIKD xenografted tumors that were treated with gemcitabine or PBS. **p* < 0.05, ***p* < 0.01 indicate statistical significance of TGF-βRII-KD compared with the NT control.
