# Supplementary material for: TGF-βRII Knock-down in Pancreatic Cancer Cells Promotes Tumor Growth and Gemcitabine Resistance. Importance of STAT3 Phosphorylation on S727
Source: Cancers (Basel). 2018 Jul 31;10(8):254. doi: 10.3390/cancers10080254 (PMC6116183; doi:10.3390/cancers10080254)
Supplement: Supplementary file 1 [file cancers-10-00254-s001.pdf]

cancers

MDPI

TGF-βRII Knock-Down in Pancreatic Cancer Cells Promotes Tumor Growth and Gemcitabine Resistance. Importance of STAT3 Phosphorylation on S727

Vincent Drubay , Nicolas Skrypek, Lucie Cordiez, Romain Vasseur, Céline Schulz, Nihad Boukrout, Belinda Duchêne, Lucie Coppin, Isabelle Van Seuningen and Nicolas Jonckheere

Supplementary Material

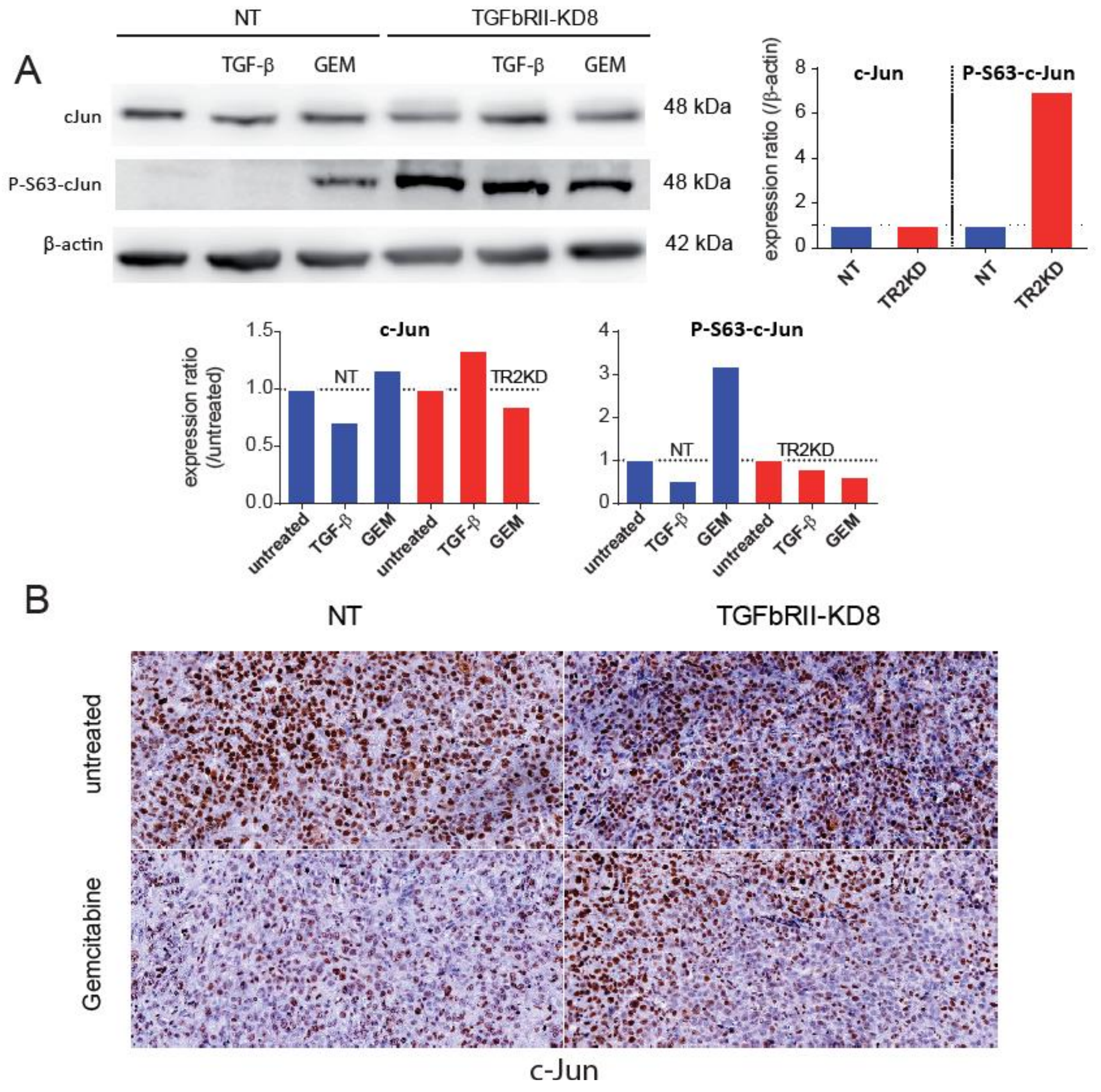

Figure S1. *Cont.*

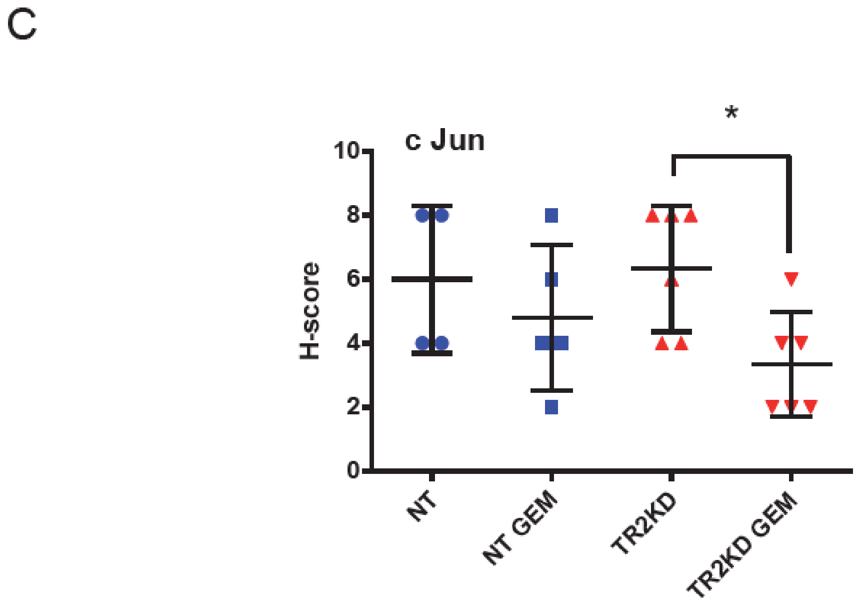

Figure S1. TGF-βRII knockdown promotes c-Jun S-63 phosphorylation in CAPAN-2 cells. (**A**) c-Jun, phospho-S63 c-Jun and β-actin expression was analyzed by western blotting. Bands intensities were quantified by densitometry and ratios (KD vs. NT or treated/untreated) are indicated in the graphs. Expression in NT (for TGF-βRIIKD) or untreated (for gemcitabine/TGF-β) cells was arbitrarily set to 1. (**B**) IHC analysis of c-Jun on extracted xenografted NT and TGF-βRIIKD tumors. (**C**) IHC staining was scored in NT and TGF-βRIIKD xenografted tumors that were treated with gemcitabine or PBS. * $p < 0.05$ indicate statistical significance of TGF-βRII-KD compared with the NT control.

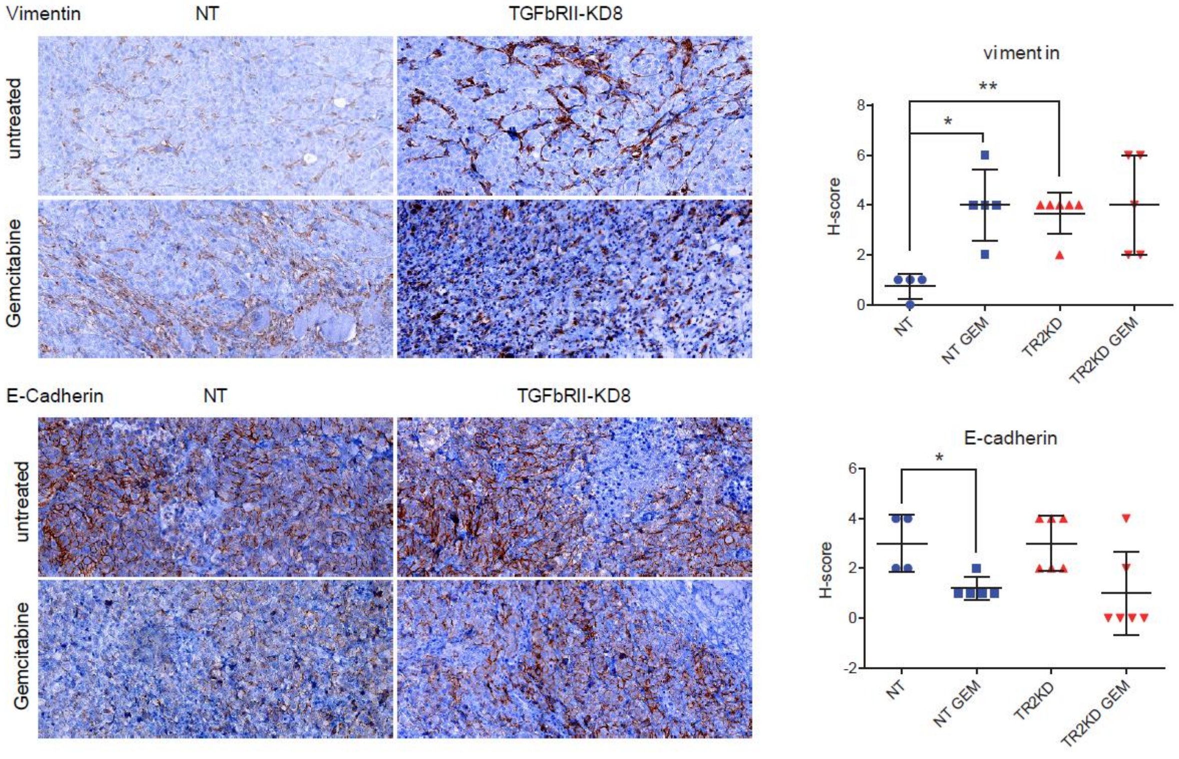

Figure S2. TGF-βRII knockdown promotes partial EMT-like phenotype. IHC analysis of E-cadherin and vimentin on extracted xenografted NT and TGF-βRIIKD tumors. IHC staining was scored in NT and TGF-βRIIKD xenografted tumors that were treated with gemcitabine or PBS. *$p < 0.05$, **$p < 0.01$ indicate statistical significance of TGF-βRII-KD compared with the NT control.
